# Circ_PIAS1 Promotes the Apoptosis of ALV-J Infected DF1 Cells by Up-Regulating miR-183

**DOI:** 10.3390/genes14061260

**Published:** 2023-06-14

**Authors:** Ting Yang, Lingling Qiu, Shihao Chen, Zhixiu Wang, Yong Jiang, Hao Bai, Yulin Bi, Guohong Chen, Guobin Chang

**Affiliations:** 1 Key Laboratory for Animal Genetics & Molecular Breeding of Jiangsu Province, College of Animal Science and Technology, Yangzhou University, Yangzhou 225009, China; yt1144736744@163.com (T.Y.); better_11qiu@163.com (L.Q.); wangzx@yzu.edu.cn (Z.W.); jiangyong12126@163.com (Y.J.); ylbi@yzu.edu.cn (Y.B.); ghchen2019@yzu.edu.cn (G.C.); 2 Institute of Epigenetics and Epigenomics, College of Animal Science and Technology, Yangzhou University, Yangzhou 225009, China; mrrchen@yzu.edu.cn; 3 Joint International Research Laboratory of Agriculture and Agri-Product Safety, the Ministry of Education of China, Institutes of Agricultural Science and Technology Development, Yangzhou University, Yangzhou 225009, China; bhowen1027@yzu.edu.cn

**Keywords:** circRNA, miRNA, cell apoptosis

## Abstract

(1) Background: circRNAs are closed circular molecules with covalent bonds generated by reverse shearing, which have high stability and have different manifestations in different tissues, cells, or physiological conditions and play important roles in various disease processes and physiological processes. In addition, circ_PIAS1 has been screened out and verified, and the bioinformatics analyzed in previous studies. In this study, we investigated the function of circ_PIAS1 and studied its role in ALV-J infection to provide a basis for the role of circRNA in ALV-J infection. (2) Methods: the effect of circ_PIAS1 on apoptosis during ALV-J infection was studied by flow cytometry and detection of apoptotic gene expression, and miR-183 was screened by a biotin-labeled RNA pull-down technique. After overexpression and inhibition of miR-183, the effect of miR-183 on apoptosis in the process of ALV-J infection was studied by flow cytometry and detection of apoptotic gene expression. (3) Results: after overexpression of circ_PIAS1, flow cytometry and apoptotic gene expression showed that circ_PIAS1 promoted apoptosis. The results of RNA pull-down showed that 173 miRNAs could bind to circ_PIAS1, and circ_PIAS1 up-regulated the expression of miR-183. On the other hand, the same results were obtained whether miR-183 was overexpressed or inhibited that miR-183 affected ALV-J infection by promoting cell apoptosis. (4) Conclusions: circ_PIAS1 up-regulated the expression of miR-183 and influenced ALV-J infection by promoting cell apoptosis.

## 1. Introduction

Circular RNAs (circRNAs) are a class of non-coding circular RNA molecules that are widely present in animal and plant cells and can regulate gene expression. CircRNAs were discovered in viruses in the 1970s [1]. However, for a long time, they have been considered to be by-products of splice-mediated splicing errors or intermediates of escaping intron lassos [2,3], and they have not received much attention. In recent years, the research on circRNA has made people re-understand the function of RNA, and the special structural characteristics of circRNA make it have specific biological functions. Current studies have found that circRNA can regulate the expression of host genes. It interacts with RNA-binding proteins to regulate translation processes, plays a role in cis-transcriptional regulation, and even regulates variable splicing. However, the relatively mature research is that circRNAs can act as competitive endogenous RNAs (ceRNAs), competing with mRNAs for the target binding sites of miRNAs to regulate gene expression. CircRNA 28313 relieves miR-195A-mediated inhibition of CSF1(Colony-Stimulating Factor 1) through the ceRNA mechanism and regulates osteoclast differentiation in bone marrow monocytes/macrophages [4]. Current studies have found that in addition to some proteins, miRNAs, and lncRNAs, circRNAs are also important “weapons” for cells to resist virus invasion and subsequent damage [5,6]. Studies have found that the expression level of circ_RNF13 in tumor tissues of patients with hepatitis B virus-associated hepatocellular carcinoma is significantly increased, and circ_RNF13 can inhibit tumor growth and invasion by combining with miR-424-5p to promote the expression of transcription factor TGIF2 [7].

MiRNAs are a class of single-stranded non-coding small molecule RNAs with a length of about 22 nucleotides, which play a role by inhibiting the transcription of target genes [8] or degrading the mRNA of target genes [9]. MiRNAs are widely involved in the regulation of cell growth, apoptosis, tumorigenesis, and viral infection [10,11]. By regulating the expression of viral target genes, miRNAs affect the natural course and pathological and physiological processes of diseases and have become a hot topic in the study of viral infection mechanisms. Song L [12] reported inhibition of replication of miR-323, miR-491, and miR-654 of the influenza A H1N1 virus by binding to the same conserved region of the PB1(Polar Body 1) gene. In addition, researchers examined changes in miRNA abundance in IAV-infected (Influenza A viruses) A549 cells. Differentially expressed miRNAs were identified, and it was found that the up-regulated expression of miR-203 was positively correlated with increased type I interferon response and DNA demethylation induced by IAV [13]. Several studies have demonstrated that miR-183 plays a role in a variety of cancers. In colorectal cancer, miR-183 inhibits HCT116 and HT29 cell death by regulating autophagy and apoptosis [14]. MiR-183 exerts tumor-promoting effects in ovarian cancer (OC) by affecting OC cell proliferation, migration, invasion, and apoptosis [15]. In bladder cancer (BC), miR-183-5p regulates BC cell apoptosis by targeting PNPT1(Polyribonucleotide nucleotidyl transferase 1) and BMF(Bcl2 modifying factor) to inhibit the outer mitochondrial membrane permeability of BC cells [16].

Circ_PIAS1 was obtained from the transcriptomic results of spleen tissues infected with ALV-J(Subgroup J avian leukemia virus) in previous studies, and circ_PIAS1 has been identified and bioinformatics analysis [17]. This study investigated the role of circ_PIAS1 in ALV-J infection, which provided a basis for studying the role of circRNA in ALV-J infection.

## 2. Materials and Methods

### 2.1. Cell Culture

DF-1 cells, a transmissible chicken fibroblast cell line, were obtained from American Type Culture Collection (ATCC). They were cultured in DMEM supplemented with 10% FBS (Fetal Bovine Serum) at 37 °C with an atmosphere of 5% CO_2_ and 60–70% relative humidity.

### 2.2. Vectors Construction

Primers were designed to amplify the full length of circ_PIAS1 by Nanjing Vazyme Biotechnology’s online software (https://crm.vazyme.com/cetool/singlefragment.html, URL (accessed on 10 February 2022) and synthesized by Beijing Tsingke Biological Technology synthesis (Table 1). The full-length fragment of circ_PIAS1 was amplified using the DF-1 cDNA as a template using the primers in Table 1, and then the target fragment obtained was recovered using the FastPure Gel DNA Extraction Mini Kit (Vazyme, Nanjing, China). Finally, the target fragment was combined with the linearized vector (pcDNA3.1/PMIR-REPORT) using the ClonExpress^®^ Ultra One Step Cloning Kit (Vazyme, Nanjing, China) for homologous recombination. The selected constructed vector was used as a template for PCR amplification, and Sanger sequencing was performed to compare the sequence with the target fragment, and a successful comparison indicated that the vector was constructed successfully.

### 2.3. ALV-J Infected DF-1 Cells

DF-1 cells were inoculated into 6-well plates. When the degree of cell fusion reached 40%, the diluted ALV-J venom (MOI 5) was added to the medium. After 2 h, the medium was changed into 1% medium (DMEM supplemented with 1% FBS), and transfection experiments (part 2.5 and part 2.6) according to the instructions of Lipofectamine™ 2000 were performed 12 h later(ALV-J JS09GY3 strains were donated by Professor Aijian Qin of Yangzhou University). Cells were harvested after 36 h for subsequent assays.

### 2.4. Western Blot

After ALV-J infected DF-1 cells for 48 h, the cells were collected, and then the cells were lysed with RIPA and the supernatant was collected. The protein concentration was then measured using a BCA protein concentration assay kit (Beyotime, Shanghai, China), and finally, a western blot analysis was performed.

A 12% sodium dodecyl sulfate-polyacrylamide (SDS) gel (GenScript, Nanjing, China) was used for protein separation. Then, proteins were transferred onto a polyvinylidene difluoride (PVDF) membrane (Polyvinylidene Fluoride) following 1-h blocking with 5% defatted milk. Then, primary antibodies were added and incubated at 4 °C overnight. After washing with tris-buffered saline tween (TBST), secondary antibodies were added for 1-h incubation at RT. Primary antibodies, including Mouse anti-gp85, JE9, donated by Professor Aijian Qin of Yangzhou University, and Rabbit anti-β-Actin were purchased from Novus Biologicals (Englewood, CO, USA). Secondary antibodies, including HRP Goat Anti-Mouse IgG(H+L), purchased from ABclonal (Wuhan, China), and Goat pAb to Rb IgG (HRP), purchased from Abcam (Cambridge, UK).

### 2.5. Total RNA Extraction, cDNA Synthesis, and qRT-PCR

DF-1 cells to be transfected were seeded in a 6-well cell culture dish. The pcDNA3.1, 3.1-circ_PIAS1, mimic NC, miR-183 mimic, inhibitor NC, and miR-183 inhibitor were transfected into ALV-J infected DF-1 cells according to part 2.3, respectively. A set of three biological repetitions were performed for each group. Cells were harvested, and the total RNA was extracted using TRIzol Reagent and stored at −80 °C. MiRNA cDNA was synthesized using a miRNA 1st strand cDNA synthesis Kit (by stem-loop) (Vazyme, Nanjing, China), and then the expression of miR-183 in cells was detected by miRNA Universal SYBR qPCR Master Mix (Vazyme, Nanjing, China). The cDNA synthesis of genes was performed using HiScript III RT SuperMix for qPCR (+gDNA Wiper) (Vazyme, Nanjing, China), and the expression levels of related genes in tissues and cells were detected using the ChamQ Universal SYBR qPCR Master Mix (Vazyme, Nanjing, China). QRT-PCR was performed in the QuantStudio 5 Real-Time PCR instrument. Primer sets were designed with Primer 5.0 (Table 2) and synthesized by Tsingke Biotech (Beijing, China), using chicken GAPDH and U6 as reference genes. All assays were run in triplicate. Expression levels were quantified using the 2^−ΔΔCT^ method.

### 2.6. Cell Apoptosis Assay

Apoptosis was determined using an Annexin V-FITC/PI apoptosis detection kit according to the manufacturer’s protocol (Vazyme, Nanjing, China). The pcDNA3.1, 3.1-circ_PIAS1, mimic NC, miR-183 mimic, inhibitor NC, and miR-183 inhibitor were transfected into ALV-J infected DF-1 cells according to part 2.3, respectively. A set of three biological repetitions were performed for each group. Cells were harvested, washed twice with ice-cold PBS, and evaluated for apoptosis by double staining with FITC conjugated Annexin V and propidium iodide (PI) in binding buffer for 30 min using Flow Cytometry (FACSAria SORP, BD, San Jose, CA, USA).

### 2.7. Dual-Luciferase Reporter Assay

DF-1 cells were seeded in a 24-well cell culture dish. When the degree of cell fusion is about 70–80%, they were co-transfected with PMIR-REPORT or PMIR-circ_PIAS1 and miR-183 to mimic or mimic NC (GenePharme, Suzhou, China). After 48 h of transfection, the luciferase activity was detected on the Multi-mode micropore detection system (EnSpire, PerkinElmer, Waltham, MA, USA) according to the instructions of the double luciferase detection kit (Vazyme, Nanjing, China).

### 2.8. RNA Pull-Down

For a biotin-coupled RNA pull-down assay, the cells were collected and handed over to a biological company (Ribobio, Guangzhou, China). The experimental procedure follows: Step 1: prepare lysed cell samples. Prepare DF-1 cells in the order of 5 × 10^7^ cells, trypsin digestion, centrifuge at 1000× *g* for 3 min, discard the supernatant, wash twice with pre-chilled PBS, and centrifuge to collect the cell precipitate. Lysis Buffer (2 × 10^7^ cell miles/mL) was added to resuspend the cells, along with 4 µL of PMSF (250 mmol, 1:250 added) and 10 µL of PIC (100 mmol, 1:100 added), and vortex shaking was performed 3 times to facilitate adequate sample lysis. Centrifuge at 12,000 r/m for 10 min at 4 °C and collect the supernatant for direct use in experiments or store at −80 °C. Step 2: biotin-labeled RNA was prepared. Positive and Negative probe sequences were designed based on the circ_PIAS1 gene sequence, and then biotin-labeled probes were synthesized directly by gene synthesis. Step 3: RNA_Pull_Down experimental procedure. Add the above-lysed cell samples and RNA-RNA Hybridization Buffer in a ratio of 1:2 and mix thoroughly. Add a further 5 µL of a biotin-labeled probe to the mixture as above and mix thoroughly. Incubate at room temperature overnight. Take 50 µL of streptavidin beads, place them on a magnetic stand and let stand. Discard the supernatant. Add 500 µL of RNARNA Hybridization Buffer to the streptavidin beads, place on a magnetic stand, discard the supernatant, and repeat the wash three times. Add the sample/Biotin-RNA mixture to the streptavidin beads and incubate at room temperature for 4 h. After 4 h of incubation, place on a magnetic stand and let stand, discard the supernatant. Add 1 mL Wash buffer, place on a magnetic stand and let stand, discard the supernatant, and repeat the wash five times. Add 1 mL Trizol resuspended magnetic beads to purify enriched RNA or store at −80 °C. Step 4: purify the enriched RNA. Resuspend the magnetic beads with 150 µL Proteinase K Buffer, incubate at 55 °C for 30 min, and place on a magnetic stand to transfer the supernatant to a new sample tube. Add 400 μL phenol:chloroform:isoamyl alcohol to the sample tube, vortex shake for 15 s, and centrifuge at 14,000 rpm for 10 min. Carefully aspirate 350 μL of the upper aqueous phase, add 400 μL chloroform, vortex shake for 15 s, and centrifuge at 14,000 rpm for 10 min. Carefully aspirate 350 μL of the upper aqueous phase, add 50 μL Salt Solution I, 15 μL Salt Solution II, 5 μL Precipitate Enhancer, 850 μL anhydrous ethanol (without RNase), and mix. Keep at −80 °C overnight, centrifuge at 14,000 rpm for 30 min and discard the supernatant. Wash once with 80% ethanol, centrifuge at 14,000 rpm for 15 min and discard the supernatant. After air-drying, add 20 μL of DEPC aqueous solvent and quantify directly or store at −80 °C. Step 5: the purified RNA was sequenced for library construction to identify the miRNA species bound by circ_PIAS1. The RNApulldwon enriched RNA is passed through VAHTS Small RNA Library Prep Kit for Illumina to build miRNA libraries suitable for Illumina’s high-throughput sequencing platform, and the enriched and purified RNA is connected to a universal junction, then reverse transcribed and PCR amplified. The enriched and purified RNAs are then linked to universal connectors, reverse transcribed, amplified by PCR and purified by magnetic beads to obtain sequencing libraries suitable for the Illumina platform. In contrast, the sequencing libraries undergo strict quality control to improve the stability and reproducibility of the libraries. We can identify the miRNAs that can bind to circ_PIAS1. The sequences of the positive and negative probes of circ_PIAS1 are shown in Table 3.

### 2.9. Statistical Analysis

Statistical analysis was performed using SPSS 22.0 software (SPSS Inc., Chicago, IL, USA). Analysis of two-group comparisons using one-way ANOVA was conducted. Only when *p* < 0.05 (*), *p* < 0.01 (**), or *p* < 0.001 (***) were the data considered statistically significant. In this study, three times repeated were assayed in each experiment. All data provided in this study are presented as means ± SD (standard deviation).

## 3. Results

### 3.1. Circ_PIAS1 Promotes the Apoptosis of ALV-J Infected DF1 Cells

**Figure 1:**
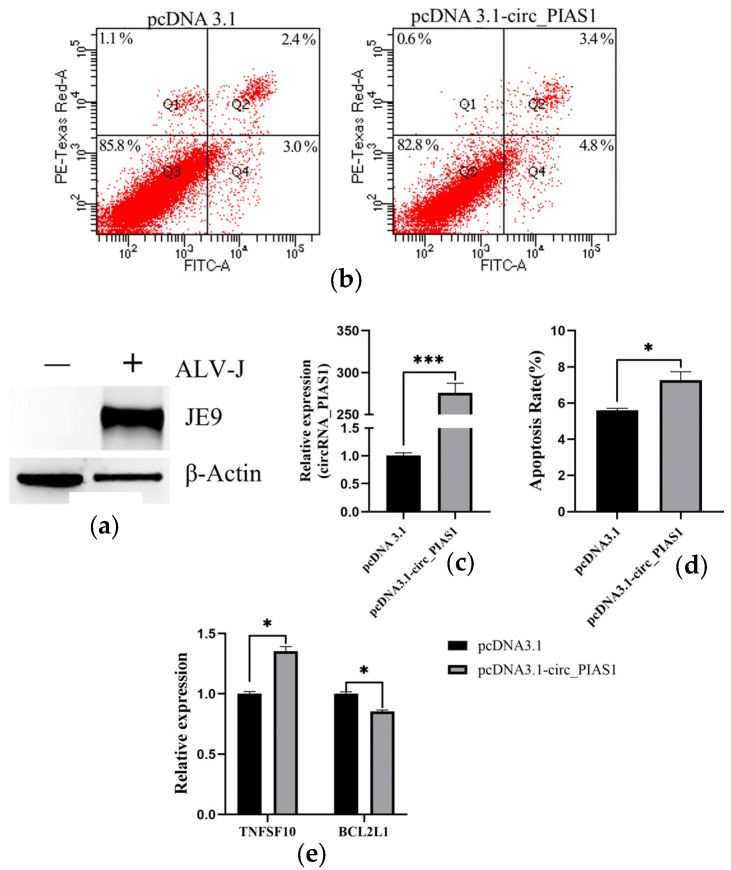
Circ_PIAS1 promotes cell apoptosis. (**a**) After the construction of the ALV-J infection model, gp85 protein expression was detected by western blot; (**b**) Cell apoptosis was detected by flow cytometry; (**c**) Circ_PIAS1 overexpression efficiency was detected by qRT-PCR; (**d**) Cell apoptosis rate was measured; (**e**) The expression of apoptosis-related genes was detected by qRT-PCR (* *p* < 0.05, *** *p* < 0.001).

In previous studies, Circ_PIAS1 was obtained from the transcriptomic results of spleen tissues infected with ALV-J. Circ_PIAS1 was identified, bioinformatics analysis was conducted, and a follow-up study was completed. First, the ALV-J infection model of DF-1 cells was constructed, and WB results showed that the ALV-J infection model was successfully constructed (Figure 1a). Then, the overexpression vector of circ_PIAS1 was constructed, and the full length of circ_PIAS1 was connected to the pcDNA3.1 vector by the homologous recombination method. QRT-PCR results showed that the overexpression vector of circ_PIAS1(pcDNA3.1-circ_PIAS1) was successfully constructed (Figure 1c). The pcDNA3.1-circ_PIAS1 was transfected into DF-1 cells infected with ALV-J, and the apoptosis was detected 48 h after transfection by flow cytometry. The results showed that circ_PIAS1 promoted cell apoptosis (Figure 1b,d). Then, the effect of circ_PIAS1 on the expression of apoptosis-related genes was detected. QRT-PCR results showed that circ_PIAS1 promoted the expression of TNFSF10(Tumor Necrosis Factor Superfamily member 10) and inhibited the expression of BCL2L1(B-cell lymphoma-2 like 1) (Figure 1e).

### 3.2. Bioinformatic Analysis of Biotin-Labeled RNA Pull-Down Analysis

**Figure 2:**
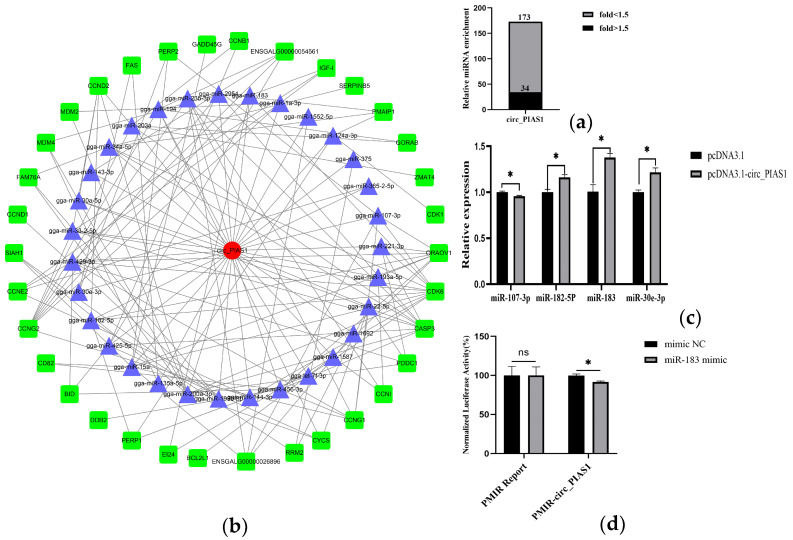
Bioinformatic analysis of biotin-labeled RNA pull-down analysis. (**a**) Statistical analysis of miRNAs that can bind to circ_PIAS1; (**b**) GO enrichment analysis of the predicted gene of 34 miRNAs with fold > 1.5; (**c**) KEGG enrichment analysis of the predicted gene of 34 miRNAs with fold > 1.5; (**d**) CeRNA regulatory network diagram of the p53 signaling pathway. (* *p* < 0.05, “ns” no significant difference).

**Figure 3:**
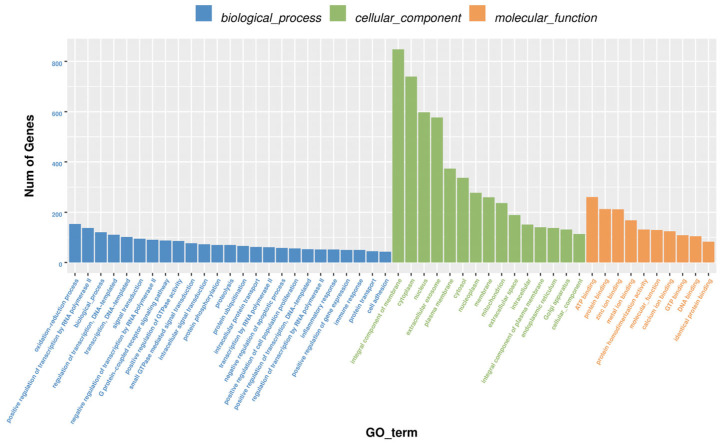
GO enrichment analysis of the predicted gene of 34 miRNAs with fold > 1.5.

**Figure 4:**
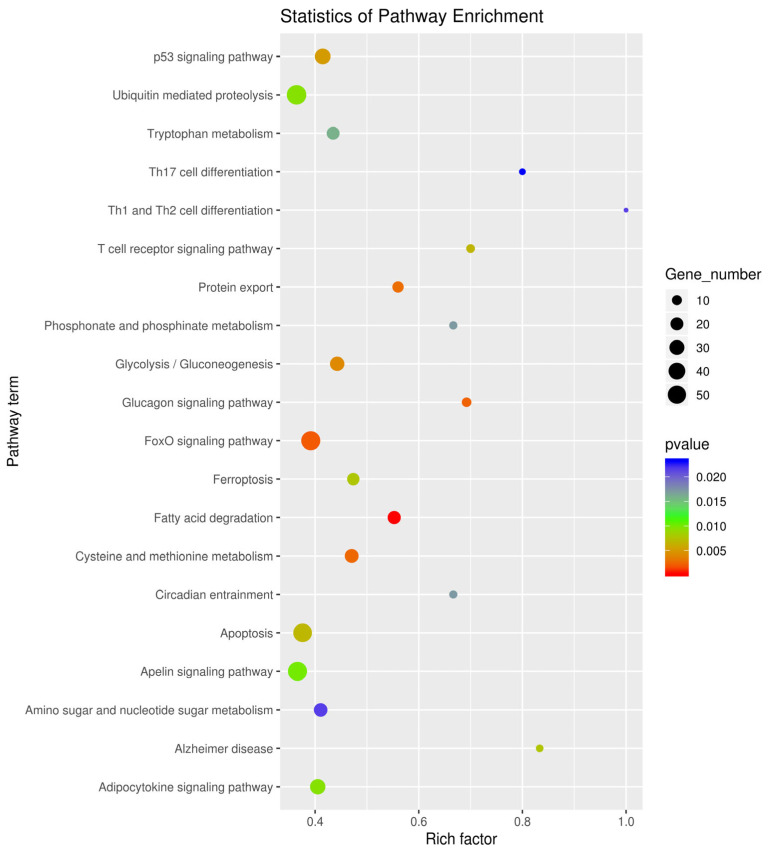
KEGG enrichment analysis of the predicted gene of 34 miRNAs with fold > 1.5.

It is reported that circRNA acts by sponging miRNA. Therefore, to analyze miRNAs that can bind to circ_PIAS1, we performed biotin-labeled RNA pull-down analysis and combined it with high-throughput sequencing. The result of biotin-labeled RNA pull-down analysis showed many miRNAs could bind to circ_PIAS1, including 34 miRNAs with fold > 1.5 and 139 miRNAs with fold < 1.5 (Figure 2a). Bioinformatic analysis was performed using the OmicStudio tools at https://www.omicstudio.cn/tool (accessed on 15 January 2023), target gene prediction, and GO (Gene Ontology) and KEGG enrichment analysis (Kyoto Encyclopedia of Genes and Genomes Pathway-based Enrichment Analysis) was performed for the 34 miRNAs with fold > 1.5 (Supplementary Table S1). GO enrichment analysis showed that the functions of the predicted gene of miRNAs were mainly integral components of the membrane, extracellular exosome, oxidation−reduction process, GTP binding, protein binding, etc. (Figure 3). KEGG enrichment analysis showed that the predicted genes of miRNAs were enriched in the FoxO signaling pathway, Apelin signaling pathway, Ubiquitin mediated proteolysis, Apoptosis, p53 signaling pathway, etc. (Figure 4). In addition, the ceRNA regulatory network of the P53 signaling pathway is demonstrated (Figure 2b). 

From these miRNAs, miR-107-3p, miR-183, miR-182-5p, and miR-30e-3p were selected and verified by quantitative PCR. The results showed that circ_PIAS1 up-regulated the expression of miR-183, miR-182-5p, and miR-30e-3p and down-regulated the expression of miR-107-3p (Figure 2c). Next, we selected the miRNA with the largest difference from the above four miRNAs for subsequent experiments and whether a targeting relationship between circ_PIAS1 and miR-183 was detected. The double luciferase reporter gene experiment results showed that miR-183 could bind to circ_PIAS1 (Figure 2d). These results also show that the biotin-labeled RNA pull-down analysis results are reliable.

### 3.3. Overexpression of miR-183 Increases the Apoptosis of ALV-J Infection DF1 Cells

**Figure 5:**
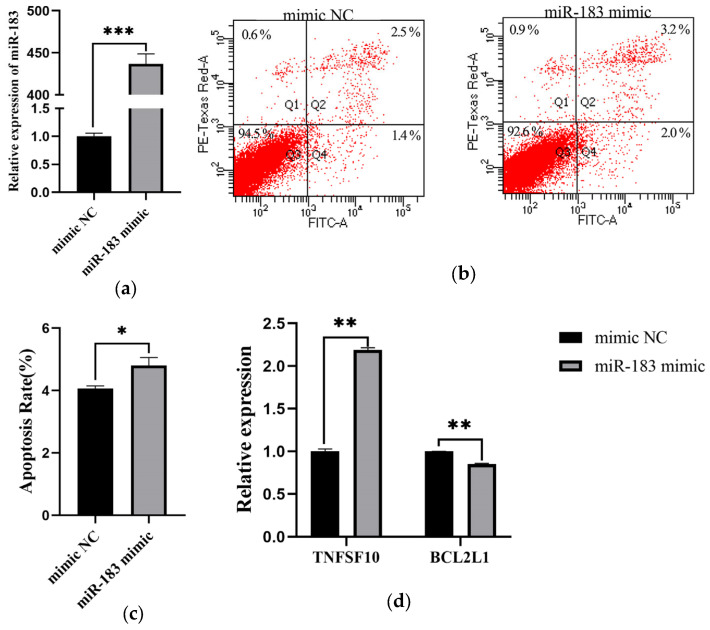
Overexpression of miR-183 increases the apoptosis of ALV-J infection DF1 cells. (**a**) The expression of miR-183 was detected by qRT-PCR; (**b**) Cell apoptosis was detected by flow cytometry; (**c**) Cell apoptosis rate was measured; (**d**) The expression of apoptosis-related genes was detected by qRT-PCR. (* *p* < 0.05, ** *p* < 0.01, *** *p* < 0.001).

It has been verified previously that circ_PIAS1 can up-regulate the expression of miR-183, and circ_PIAS1 promotes cell apoptosis, so we asked whether miR-183 also promotes cell apoptosis. First, we overexpressed miR-183 by adding miR-183 mimic to DF1 cells. QRT-PCR results showed that the expression of miR-183 was up-regulated more than 400 times (Figure 5a). The miR-183 mimic was transfected into DF-1 cells infected with ALV-J, and the apoptosis was detected 48h after transfection by flow cytometry. The results showed that miR-183 promoted DF1 cell apoptosis (Figure 5b,c). Then, the effect of miR-183 on the expression of apoptosis-related genes was detected. QRT-PCR results showed that miR-183 promoted the expression of TNFSF10 and inhibited the expression of BCL2L1(Figure 5d).

### 3.4. Inhibition of miR-183 Decreases the Apoptosis of ALV-J Infection DF1 Cells

**Figure 6:**
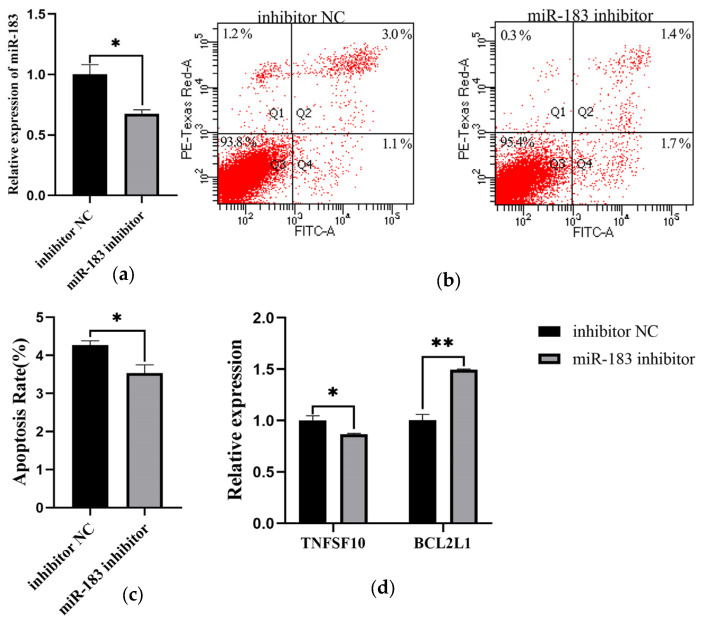
Inhibition of miR-183 decreases the apoptosis of ALV-J infection DF1 cells. (**a**) The expression of miR-183 was detected by qRT-PCR; (**b**) Cell apoptosis was detected by flow cytometry; (**c**) Cell apoptosis rate was measured; (**d**) The expression of apoptosis-related genes was detected by qRT-PCR. (* *p* < 0.05, ** *p* < 0.01).

To further explore the role of miR-183 in the process of ALV-J infection, the expression of miR-183 was inhibited (Figure 6a). The miR-183 inhibitor was transfected into DF-1 cells infected with ALV-J, and the apoptosis was detected 48h after transfection by flow cytometry. The results showed that miR-183 inhibited cell apoptosis (Figure 6b,c). Then, the effect of miR-183 on the expression of apoptosis-related genes was detected. QRT-PCR results showed that miR-183 inhibited the expression of TNFSF10 and promoted the expression of BCL2L1 (Figure 6d).

## 4. Discussion

As the role of circRNAs becomes increasingly important, these differential expressions of circRNA in different state cells have also attracted the attention of researchers. Their complex functions and mechanisms of action in viral infection often play an important biological role in the occurrence, development, and prognosis of the disease.

In previous studies, circ_PIAS1, which was highly expressed in ALV-J infected spleen tissues, has been screened, identified and bioinformatics analyzed. Next, the role of circ_PIAS1 in the infection of ALV-J was studied. Numerous studies have found that circRNAs are closely associated with apoptosis. Inhibition of circRNA-UBE2G1 expression reduced the effect of LPS on C28/I2 cell viability and apoptosis [18]. CircRNA hsa_circRNA_104348 is significantly upregulated in HCC (hepatocellular carcinoma) tissues and cells, and the circRNA hsa_circRNA_104348 may act as a competitive endogenous RNA that affects HCC cell proliferation, migration, invasion and apoptosis by targeting miR-187-3p and RTKN2 (Rhotekin2) and activating the Wnt/β-catenin pathway to promote HCC progression [19]. These results demonstrate that circRNA is associated with apoptosis. For this reason, we next investigated the effect of circ_PIAS1 on apoptosis during ALV-J infection, and our results suggest that circ_PIAS1 promotes apoptosis. In this study, both flow cytometry and quantitative PCR showed that circ_PIAS1 affected the apoptosis of ALV-J infection DF1 cells. Qiu’s results also showed that circ_2420 promoted apoptosis and affected ALV-J infection [20]. In osteoarthritis (OA), MTT(3-(4,5-dimethyl-2-thiazolyl)-2,5-diphenyl-2-H-tetrazolium bromide, Thiazolyl Blue Tetrazolium Bromide) and flow cytometry as well as western blot data elucidated that IL-1β stimulation inhibited chondrocyte proliferation and promoted apoptosis. At the same time, interference with circRNA_0092516 reversed these effects, suggesting that circRNA_0092516 inhibits chondrocyte proliferation and inflammatory factor release and promotes apoptosis [21]. In a human myeloid leukemia cell model, circRNA.0007127 associates with miR-513a-5p or CASP8 to reduce apoptosis by inhibiting CASP8 pathway activation [22]. In diabetic cardiomyopathy (DCM), cyclic RNA cerebellar degeneration-associated protein 1 antisense (Circ-CDR1as) promotes cardiomyocyte apoptosis by activating the Hippo signaling pathway through significant inhibition of mammalian sterile 20-like kinase 1 (MST1) ubiquitination levels [23]. These results are consistent with our results. Numerous studies have shown that circRNA exerts its function through sponge miRNA. CircRNA_0048211 can bind miRNA-93-5p, and BMP2 (bone morphogenetic protein 2) is the direct target of miRNA-93-5p. Meanwhile, circRNA_0048211 was negatively correlated with miRNA-93-5p and positively correlated with BMP2. In addition, the circRNA_0048211/miRNA-93-5p/BMP2 regulatory loop was responsible for regulating the expression of osteogenic-related genes, ALP (alkaline phosphatase) activity and mineralization capacity in hBMSCs (human bone marrow mesenchymal stem cells) [24]. CircRNA-UBE2G1 binds to miR-373 as competing for endogenous RNA (ceRNAs). HIF-1a (hypoxia-inducible factor-1a) may act as a target of miR-373. Furthermore, the inhibition of miR-373 expression or overexpression of HIF-1a restored the effect of circRNA-UBE2G1 downregulation on LPS-induced chondrocyte injury [18]. Immunoprecipitation and dual luciferase reporter gene assays verified that circRNA_100367 can bind to miR-217. In addition, miR-217/Wnt3 was shown to be a downstream target of circRNA_100367 in the regulation of ESCC (esophageal squamous cell carcinoma) radiosensitivity [25]. Thus, we attempted to screen miRNA that can bind to circ_PIAS1 by biotin-labeled RNA pull-down technique. Firstly, a biotin-labeled circ_PIAS1 probe was used to pull down RNA, and then miRNA library sequencing was performed on the pulled RNA to identify the types of miRNA that can bind to circ_PIAS1. The results of the RNA pull-down experiment showed that a total of 173 miRNAs could bind to circ_PIAS1. In addition, four miRNAs were selected for quantitative PCR verification, and it was found that circ_PIAS1 regulated the expression of these four miRNAs. The miR-183 with the greatest fold change was selected for subsequent study, and the dual luciferase reporting experiment also confirmed the targeting relationship between circ_PIAS1 and miR-183.

In many studies, circRNA acts as ceRNA and exerts its function by relieving the inhibition of miRNA on target genes [26]. The circRNA MFACR regulates mitochondrial fission and apoptosis in the heart by directly targeting and downregulating miR-652-3p, which in turn prevents mitochondrial division and cardiomyocyte death by inhibiting the translation of MTP18 (mitochondrial protein 18) [27]. Circular RNA CDR1 (cerebellar degeneration-related protein 1) acts as a miR-7 sponge, leading to upregulation of growth differentiation factor (GDF) 5 and phosphorylation of Smad1/5/8 and p38 mitogen-activated protein kinase (p38 MAPK), which in turn affects osteogenic differentiation [28]. Zhang’s study verified the effect of the sponge function of circ-Vav3 on its downstream genes. The sponge function of circ-Vav3 removes the effect of ga- mir -375 on the target gene YAP1 (Yes-associated protein 1), increases the expression level of YAP1, affects epithelial-mesenchymal transition (EMT) markers to promote tumorigenesis, and thus induces epithelial-mesenchymal transition (EMT) [29]. Chicken circSVIL functions as a miR-203 sponge and upregulates the mRNA levels of c-JUN and MEF2C. In chickens, circSVIL promotes the proliferation and differentiation of myogenic cells and antagonizes the function of miR-203 [30]. In this study, circ_PIAS1 up-regulated the expression of miR-183 with the greatest fold change in screening, which was inconsistent with the results of many studies. In their research, circRNA reduced the expression of miRNA [7,8,9,10]. However, circRNA reducing the expression of miRNA is only one of the mechanisms, and studies have also shown that circRNA acts by up-regulating miRNA expression. Li W observed that circCSNK1G3 was highly expressed in renal cell carcinoma tissues, and the pro-tumor effect of circCSNK1G3 was achieved by up-regulation of miR-181 [31]. Han K found that circLONP2 recruits DiGeorge syndrome key region gene 8 (DGCR8) and Drosha complex in a DDX1-dependent manner, directly interacts with primary microRNA-17 (pri-miR-17), and promotes its processing. At the same time, upregulated mature miR-17-5p can be assembled into exosomes and internalized by neighboring cells, enhancing their invasiveness [32]. In addition, some studies have demonstrated that circRNAs are not only sponges for miRNAs but also have other roles, which may provide new insights into the mechanisms of circRNAs in biological regulation [33,34].

MicroRNAs (miRNAs) are widely present in cells and body fluids and can participate in regulating the behavior of immune cells and various physiological and pathological processes, such as immunity, inflammatory reactions, and organ damage. MiR-183 has been reported to affect disease progression through apoptosis. It was found that in colorectal cancer, miR-183 regulates apoptosis in colorectal cancer cells by targeting UVRAG [14], which has been shown to have anti-apoptotic activity through interaction with Bax (BCL-2-associated X) during tumor treatment [35]. In ovarian cancer, miR-183 regulates cell proliferation and apoptosis by targeting Smad4 (Recombinant Mothers Against Decapentaplegic Homolog 4) through the TGF-β/Smad4 signaling pathway [15]. In addition, several studies have found that the TGF-β/Smad4 signaling pathway controls signal transduction from the cell membrane to the nucleus and is involved in a series of cellular processes such as cell proliferation, differentiation, apoptosis, migration, and cancer development and progression [36]. In bladder cancer (BC), miR-183-5p regulates cell apoptosis by targeting PNPT1 and BMF to inhibit the outer mitochondrial membrane permeability of BC cells [16]. It was found that during MOMP(mitochondrial outer membrane permeabilization), mitochondria release PNPT1 to initiate apoptotic decay of RNA lacking the 3’ structure and affect apoptosis [37]. It has also been shown that BMF increases the rate of apoptosis in sheep granulosa cells, while TGF-β induces BMF expression through the Smad4 pathway in a variety of cells, which in turn induces apoptosis [38]. In this study, flow cytometry and apoptotic gene expression indicated that miR-183 could promote the apoptosis of ALV-J infection DF1 cells. This result is similar to previous findings that miR-183 affects the disease process by affecting apoptosis. Gong Y’s study showed that miR-183 significantly increased the proliferation of HL-7702 cells and prevented cell apoptosis after I/R injury [39].

In conclusion, in the present study, circ_PIAS1 affected apoptosis by promoting the expression of miR-183, which in turn affected the process of ALV-J infection.

## 5. Conclusions

In this study, we investigated the function of circ_PIAS1 screened in previous studies and found that circ_PIAS1 can promote the apoptosis of ALV-J infection DF1 cells. Meanwhile, we found that miR-183 can bind to circ_PIAS1 and up-regulate the expression of miR-183. In addition, miR-183 can also promote the apoptosis of ALV-J infection DF1 cells. In summary, our study reveals that circ_PIAS1 promotes the apoptosis of ALV-J infection DF1 cells by up-regulating miR-183. These results broaden our insight into the role of circ_PIAS1 in chicken ALV-J infection.

## Figures and Tables

**Table 1:** The primer sequences for vector construction.

Primer Name	Primer Sequence (5′-3′)
3.1-circ_PIAS1	F:ctagcgtttaaacttaagcttCAAATGTAATGAGCCTTAGAGTTTC
	R:aacgggccctctagactcgagCTAGGCTGGTAGGTTTTATCAGCTC
PMIR-circ_PIAS1	F:ggaaagtccaaattgctcgagCAAATGGTAATGAGCCTTAGATTTC
	R:gccagtgccaagctagcggccgcCTAGGCTTAGGTTTTATCAGCTC

Note: The underlined letters are enzyme cutting sites, and the lowercase letters are homologous arm sequences of the vector.

**Table 2:** The primer sequences for qRT-PCR.

Primer Name	Primer Sequence (5′-3′)
circRNA_PIAS1	F: CCTCACATCTGCGCTCCAC
	R: GGGTGTACGCTGGAGAGAGAT
TNFSF10	F: TGGCCGTCACCTACATCTAC
	R: TCAGCCACTCTGTCTTTGCT
BCL2L1	F: CAGGAGCTGCTAAGTGTGCT
	R: CCCGGTTACTGCTGGACATT
GAPDH	F: CGATCTGAACTACATGGTTTAC
	R: TCTGCCCATTTGATGTTGC
U6	F: GGAACGATACAGAGAAGATTAGC
	R: TGGAACGCTTCACGAATTTGCG
miR-183 Reverse transcription	GTCGTATCCAGTCAGGGTCCGAGGTATT CGCACTGGATACGACCAGTGA
miR-183	F: CGCGTATGGCACTGGTAGAAT

**Table 3:** Circ_PIAS1 biotin-labeled Positive and Negative probe sequences.

Probes	Sequences (5′-3′)
circ_PIAS1_Positive1-probe-Biotin	CATTACCATTTGCTAGGCTGGTAG
circ_PIAS1_Positive2-probe-Biotin	TTTGCTAGGCTGGTAGGTTTTATC
circ_PIAS1_Positive3-probe-Biotin	CTAAGGCTCATTACCATTTGCTAG
circ_PIAS1_Negative-probe-Biotin	CTACCAGCCTAGCAAATGGTAATG

## Data Availability

Data presented in this study are available upon request from the corresponding author.

## Supplementary Materials

The following supporting information can be downloaded at: https://www.mdpi.com/article/10.3390/genes14061260/s1, Table S1: The list of the 34 miRNAs with fold > 1.5.
